# Secondary Angle Closure Glaucoma due to Massive Subretinal and Suprachoroidal Hemorrhage in Neovascular Age–Related Macular Degeneration: Clinical Case and Literature Review

**DOI:** 10.1155/crop/2230622

**Published:** 2025-12-04

**Authors:** Kosuke Nagaoka, Shinji Makino, Satoru Inoda, Toshikatsu Kaburaki

**Affiliations:** Department of Ophthalmology, Jichi Medical University, Tochigi, Japan

## Abstract

Massive subretinal hemorrhage secondary to neovascular age–related macular degeneration (nAMD) is relatively uncommon, and reports of secondary angle closure glaucoma associated with such hemorrhage are even rarer. We report a case of a 93-year-old woman with a history of nAMD who developed this condition, along with a review of the relevant literature.

## 1. Introduction

Subretinal hemorrhage (SRH) is commonly caused by neovascular age–related macular degeneration (nAMD), including polypoidal choroidal vasculopathy (PCV), or by retinal arterial macroaneurysm. Other etiologies include pathological myopia, Valsalva retinopathy, and trauma, among others. When the hemorrhage is located beneath the macular (submacular hemorrhage (SMH)), the natural course of visual acuity (VA) is generally poor. SRH is reported to occur in approximately 12% of nAMD patients [1]; however, massive SRH extending beyond the vascular arcade is relatively rare, occurring in only about 3% of cases [2]. Another report suggests that the incidence of SMH with loss of vision in treated nAMD was 0.46% in a 10-year observational study [3].

Suprachoroidal hemorrhage (SCH) refers to the accumulation of blood within the suprachoroidal space. Although uncommon, it is a serious complication known to occur during intraocular surgery or as a result of ocular trauma. Spontaneous suprachoroidal hemorrhage (SSCH) is thought to be associated with factors such as choroidal fragility and a predisposition to hemorrhage. Older age, hypertension, and oral anticoagulant medications have been implicated, as have ophthalmologic conditions such as high myopia, nAMD, and choroidal malignancies [4]. In cases of heavy bleeding, SRH and SSCH may be intermingled and difficult to clearly distinguish.

Massive SRH associated with nAMD is known to have a significantly poor visual prognosis on its own [5] but may additionally be associated with acute angle closure glaucoma, and such tragic cases have been reported since 1982 [6–10].

Here, we report a rare case of massive SRH secondary to nAMD that led to secondary angle closure glaucoma (SACG). Additionally, we conducted a review of previously reported cases in the literature.

## 2. Case Presentation

A 93-year-old woman presented to a local ophthalmology clinic with a complaint of pain in her left eye. Although elevated IOP was noted, the condition was initially considered manageable with observation. The following day, she developed a headache and was referred to the internal medicine department at our hospital by her local primary care physician. A head computed tomography (CT) scan revealed intraocular hemorrhage in the left eye, leading to a referral to our ophthalmology department. Her systemic medical history included hypertension, diabetes mellitus, and use of edoxaban tosilate hydrate at a dose of 15 mg/day.

**Figure 1:**
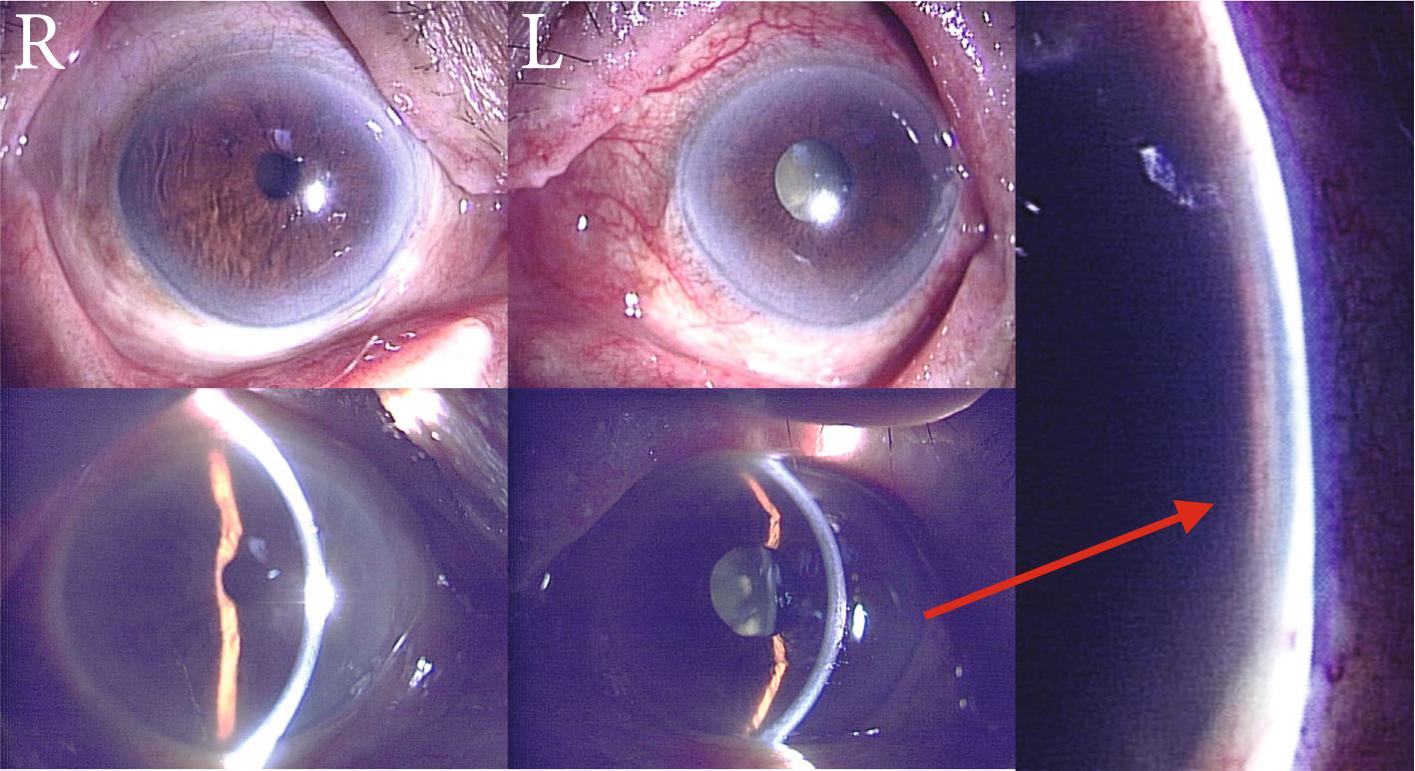
The arrow indicates that there is almost no gap between the cornea and iris near the limbus of the left eye, and the anterior chamber is very narrow.

**Figure 2:**
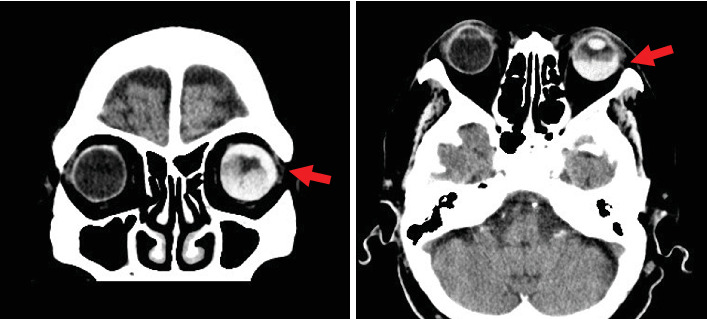
A head CT scan revealed a high absorption area (red arrow) in the left eye, suggesting intraocular hemorrhage.

**Figure 3:**
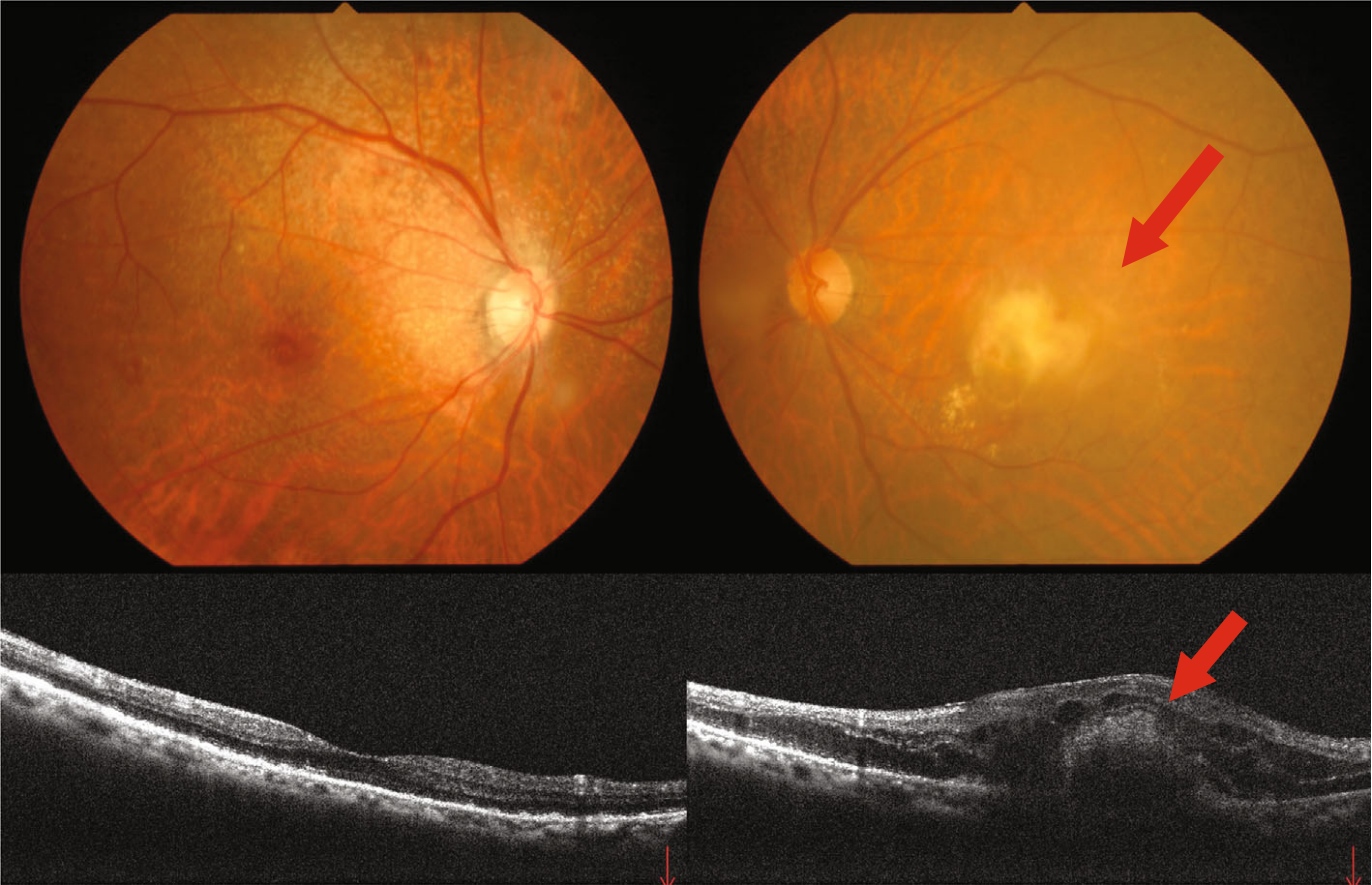
Fundus camera and OCT photograph taken 11 years ago. The arrow indicates the scar from the submacular hemorrhage.

At the initial ophthalmologic examination, her best-corrected VA was 20/100 in the right eye and hand motion (HM) in the left eye. The IOP was 16 mmHg in the right eye and 45 mmHg in the left eye. The right eye had undergone cataract surgery with intraocular lens (IOL) implantation 11 years earlier. Slit lamp examination of the left eye revealed conjunctival injection, a shallow anterior chamber (anterior chamber depth of 1.98 mm, van Herick Grade 1), and cataract (Figure 1). The axial length of the left eye was 24.17 mm. Fundus examination of the left eye was not possible due to dense vitreous hemorrhage. A head CT scan revealed a hyperdense area within the left globe, consistent with intraocular hemorrhage (Figure 2). The patient had a history of nAMD in the left eye, including a SMH that occurred 11 years earlier (Figure 3).

**Figure 4:**
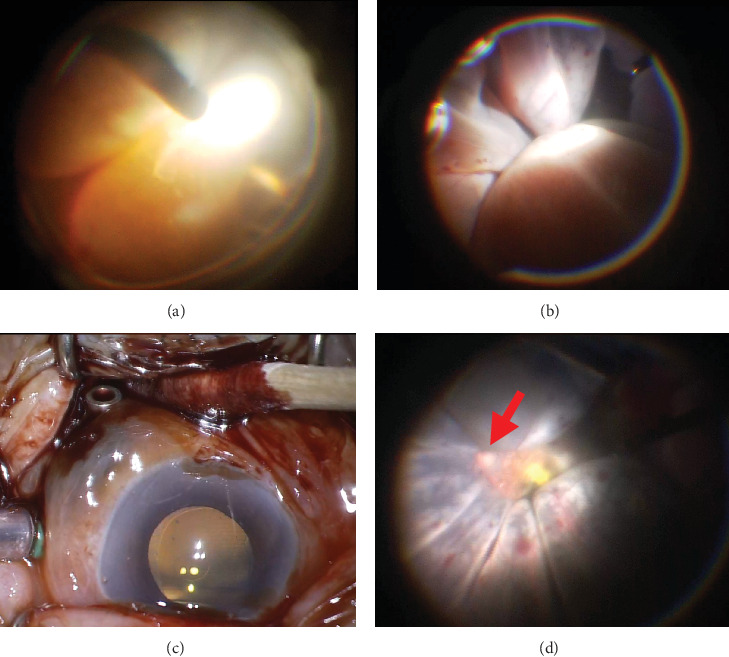
Intraoperative findings. (a) A kissing choroidal detachment is observed posterior to the vitreous hemorrhage. (b) A clearer view of the kissing choroidal detachment after removal of the vitreous hemorrhage. (c) Subretinal hemorrhage is being drained through a 27-gauge chandelier port inserted into the sclera. (d) The optic disc (arrow) and macular region became visible following drainage of the hemorrhage.

She was diagnosed with vitreous hemorrhage and secondary acute angle closure glaucoma in the left eye. On the same day, she underwent phacoemulsification with IOL implantation combined with 25-gauge pars plana vitrectomy (PPV). The cataract surgery was completed without complications. The vitreous cavity was largely absent and vitreous hemorrhage and SRH and SCH with retinal “kissing” were observed (Figure 4a,b). Using a 27-gauge chandelier port, a sclerotomy was performed, allowing the evacuation of blood from the subretinal and suprachoroidal spaces (Figure 4c). After the release of the kissing configuration, the arcade region of the retina became visible (Figure 4d), and the surgery was completed. Intraoperative findings in the macular area confirmed nAMD.

**Figure 5:**
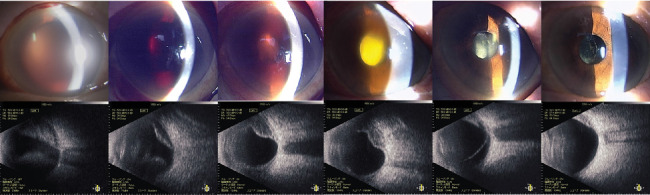
Postoperative progression of slit photographs (top) and B-mode images (bottom). The anterior chamber hemorrhage and subretinal hemorrhage are slowly being absorbed. From left to right: slit photographs and B-mode images taken 1 day, 1 week, 1 month, 3 months, 6 months, and 12 months after surgery.

Postoperatively, anterior chamber hemorrhage was observed. However, the IOP decreased to approximately 20 mmHg, and the ocular pain resolved. The anterior chamber hemorrhage, SRH, and SCH gradually resolved (Figure 5).

**Figure 6:**
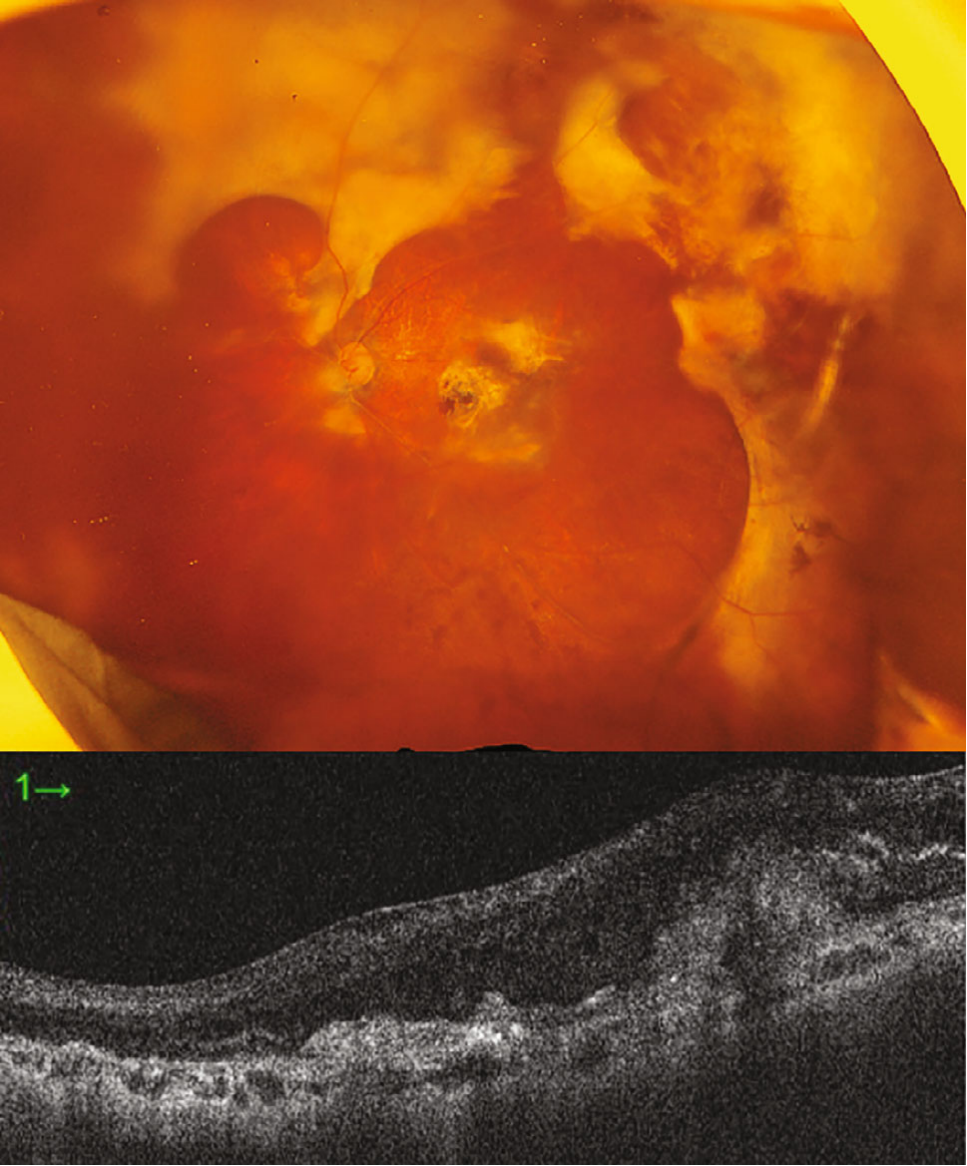
Extensive retinal damage and whitening of the retinal arteries following subretinal hemorrhage.

At 3 weeks postoperatively, the VA in the left eye was no light perception (NLP). By 3 months postoperatively, the IOP in the left eye stabilized at 3–5 mmHg. However, the shape of the globe was preserved, and no ocular pain was noted up to 12 months postoperatively. At 12 months postoperatively, wide-field fundus photography and OCT revealed extensive retinal damage and ischemia (Figure 6).

## 3. Methods

Secondary glaucoma associated with massive intraocular hemorrhage has been reported since 1982 [6–10], but detailed characteristics remain unclear. We conducted a review of reported cases of SACG associated with massive SRH or SSCH. Data were retrieved by performing a PubMed search using combinations of the following keywords: “acute glaucoma,” “secondary glaucoma,” “angle closure glaucoma,” “massive subretinal hemorrhage,” “suprachoroidal hemorrhage,” and “hemorrhagic retinal detachment.” Among the search results, we included cases with detailed records of age, sex, VA, IOP, and other relevant clinical findings. Only cases reporting abnormally elevated IOP (> 35 mmHg) alongside a shallow anterior chamber or angle closure were selected. The reviewed reports spanned the period from 1997 to 2023.

## 4. Results

A total of 30 cases with detailed preoperative and postoperative data, including treatment modalities, were identified and summarized in Table 1 [11–35].

The mean age of the patients was 72.7 ± 14.3 years, and 18 cases (60.0%) were female. nAMD was the underlying disease in 17 cases (56.7%), and IOL had been implanted in seven eyes (23.3%). Hypertension and anticoagulant use were each reported in 17 cases (56.7%) and 16 cases (53.3%). Preoperative VA ranged from NLP to 20/32. Excluding four cases with unreported data, among the 26 cases with recorded VA, 24 eyes (92.3%) had counting fingers (CFs) or worse, and 21 eyes (80.8%) had HM vision or worse. The mean IOP was 58.0 ± 10.2 mmHg. Excluding two cases with missing data, final vision of HM or worse was observed in 24 eyes (85.7%).

The 30 cases were divided into the nAMD and non-nAMD groups in Table 2.

The nAMD group consisted of 17 cases, with a mean age of 76.7 ± 10.4 years and nine females (52.9%). Both initial and final VA were HM or worse in all cases (100%). The non-nAMD group consisted of 13 cases, with a mean age of 67.4 ± 17.2 years and 9 females (69.2%). In this group, seven eyes (58.3%) had an initial VA of HM or worse, and eight eyes (66.7%) had a final VA of HM or worse. These findings suggest that the nAMD group tended to be older and had poorer visual outcomes.

There is no established treatment protocol, but conservative treatments including intravenous drip, oral medication, and eye drops have been utilized. In addition, interventions such as peripheral iridotomy and laser iridotomy, hematoma drainage via scleral incision, vitrectomy, and enucleation or evisceration have been performed. Three cases without nAMD who underwent PPV had good visual outcomes.

## 5. Discussion

This case involves an elderly female patient with a history of SMH secondary to nAMD. She developed a massive SRH so extensive that the vitreous cavity was obliterated, and it was accompanied by SACG. The patient was on antihypertensive and anticoagulant medications. While therapeutic interventions alleviated ocular pain and preserved the globe, complete loss of visual function occurred.

The causes of SACG can be classified as follows: (1) pupillary block; (2) direct angle closure due to forward movement of the lens (e.g., lens dislocation or bulging lens); (3) forward displacement of posterior segment tissues (e.g., in cases of microphthalmia, pan-retinal photocoagulation, intraocular tumors, posterior scleritis, ciliochoroidal detachment caused by conditions such as Vogt–Koyanagi–Harada disease, malignant glaucoma, intraocular tamponade, massive intraocular hemorrhage, or retinopathy of prematurity); and (4) peripheral anterior synechia unrelated to anterior chamber depth (e.g., due to neovascularization, iridocorneal endothelial syndrome, uveitis, surgery, or trauma), as outlined in Glaucoma Treatment Guidelines, fifth edition in Japan [36].

In the present case, the mechanism of SACG was most likely the forward displacement of the lens secondary to massive hemorrhage in the posterior segment, which corresponds to category (3). This is further supported by the absence of a short axial length. Similar mechanisms have been proposed in previously reported cases [17, 18, 22, 23, 30, 32, 33, 35]. However, SACG caused by a similar mechanism has also been observed in eyes with IOL. This suggests that even in the absence of a natural lens, significant posterior hemorrhage can lead to anterior chamber collapse and subsequent SACG. Therefore, cataract surgery, a common treatment and preventative treatment for primary angle closure glaucoma, is likely to have limited effectiveness in such cases. To prevent SACG in similar scenarios, it is critical to control the activity of nAMD and to minimize the occurrence of massive hemorrhages. The reported frequency of SRH due to nAMD varies among studies [1–3, 37]. However, in cases of PCV, the frequency of massive SRH is reported to be three times higher than in typical nAMD [38]. A study has also suggested that combination therapy with photodynamic therapy may reduce this risk [39].

This case involved a woman in her 90s with a history of nAMD, hypertension, diabetes mellitus, and antiplatelet medication use, all of which are known predisposing factors previously reported. Based on the clinical history, the second episode of ocular pain is consistent with elevated IOP due to SACG. However, the ocular pain experienced on the previous day was judged as not requiring treatment by the initial physician, suggesting that the IOP elevation at that time might not have been severe. As the patient initially presented to the emergency department without a referral letter, the details are uncertain. Nevertheless, it is possible that the initial ocular pain was caused by choroidal hemorrhage rather than elevated IOP. The cause of vision loss was presumed to be a massive SRH, as evidenced by marked retinal damage, whitening of the retinal arteries, and a normal appearance of the optic disc. In elderly patients with nAMD, it is particularly crucial to assess systemic conditions and confirm the use of anticoagulant medications. For patients at high risk of hemorrhage, regular fundus examinations, continued intravitreal anti-vascular endothelial growth factor therapy as needed, and, if feasible, dose adjustment of anticoagulant medications should be considered.

## Figures and Tables

**Table 1:** Review of 30 patients with SACG developed from massive subretinal hemorrhage.

**Reference (year)**	**Age**	**Sex**	**Eye**	**MH**	**OH**	**Anticoagulant**	**Initial VA**	**Initial IOP**	**Management**	**Final VA**
Poller and Hesse [11], 1997	72	F	R	Platelet dysfunction	AMD	NA	NA	50-80	Sclerotomy, evisceration	—

Chorich et al. [12], 1998	66	F	R	HTN, AMI	Moderate myopia	TPA	HM-LP	60	Medications, LI, PI	LP

Alexandrakis et al. [13], 1998	86	F	R	HTN, CAF	AMD	Warfarin	HM	49	LI, medications	LP

Wong [14], 1999	76	M	R	HTN, IHD, DM	IOL	Heparin	CF	60	Medications	NLP

Chen et al. [15], 2001	57	M	R	HTN, CBE, DM	AMD	None	HM	50	Medications	NLP
	78	M	L	CAD	AMD	None	NLP	50	Sclerotomy	NLP
	55	M	R	HTN	AMD	None	NLP	42	Sclerotomy	HM
	67	F	L	HTN, DM, VHC	AMD	None	NA	70	Sclerotomy	NA

Neudorfer et al. [16], 2002	84	F	L	IHD	IOL	Enoxaparin	CF	70	Medications	20/100

Knox and Johnston[17], 2002	81	F	R	AF	AMD	Warfarin	LP	36	Iridotomy	NLP

Yang et al. [18], 2003	84	M	R	HTN	AMD	None	HM	43	Sclerotomy	NLP
	66	F	L	MVR	AMD	Warfarin	LP	40	Sclerotomy	NLP

Kamizuru and Hideo [19], 2005	76	M	L	HT	AMD	None	HM	70	Medications, LI, PEA+IOL + PPV	NLP

Barsam et al. [20], 2006	86	F	R	DM, IHD	IOL, Glaucoma	Aspirin	NLP	> 50	Medications	HM

Saeed et al. [21], 2007	27	F	L	DM, hemodialysis	IOL, DR	TPA	CF	48	PPV, SO	6/12

Lee et al. [22], 2007	81	F	L	HTN,	None	Aspirin, plavix	NLP	58	Enucleation	—

Chandra et al. [23], 2009	84	M	L	HTN, CBS, PE	AMD, glaucoma	Warfarin	NLP	44	Medications	NLP

Liu et al. [24], 2009	80	M	R	HTN	AMD	None	LP	75	Medications, LI, PPV + sclerotomy+SO	LP

Fukuchi et al. [25], 2009	46	F	L	HTN	NA	NA	NA	55	Medications	NA

Chen et al. [26], 2009	86	M	R	IHD, DVT	IOL, AMD, glaucoma	Aspirin	NLP	45	Sclerotomy, evisceration	—

Lim et al. [27], 2011	75	F	L	AA	Hypermetropic	None	HM	70	Paracentesis, enucleation	—

Andreatta et al. [28], 2016	90	F	L	AF	AMD	Warfarin	HM	55	Medications	LP

Hisao et al. [29], 2016	64	M	R	HTN, BS	None	Clopidogrel	NLP	59	Iridotomy, sclerotomy	NLP

Masri et al. [30], 2018	67	M	R	AF	Glaucoma	Warfarin	6/12	42	Medications, PEA+IOL + PPV + sclerotomy	6/24

Ecsedy et al. [31], 2018	76	F	L	HTN, DM, AR	AMD	Acenokumarol	NA	52	CPC	NLP
	77	F	R	HTN, AF	AMD	Warfarin	NLP	61	CPC	NLP

Sosuan and Domingo [32], 2019	52	F	R	HTN	NA	None	NLP	60	Medications, enucleation	—

Holbrook et al. [33], 2020	90	F	L	None	AMD, IOL	None	NLP	56	Medications	NLP

Cruz-Pimentel et al. [34], 2022	84	M	R	CML, LL	IOL	NA	LP	70	Medications, PI, operation	20/100

Ng et al. [35], 2023	69	F	L	DM, HTN, IHD, CKD	DR	Clopidogrel	20/32	56	Medications, PPV, evisceration	—

Abbreviations: AA, aortic aneurysm; AF, atrial fibrillation; AMI, acute myocardial infarction; AR, aortic regurgitation; BS, brainstem stroke; CAD, coronary artery disease; CBE, cerebrovascular episode; CBS, cardiac bypass surgery; CFs, count fingers; CKD, chronic kidney disease; CML, chronic myeloid leukemia; CPC, cyclophotocoagulation; DM, diabetes mellitus; DR, diabetic retinopathy; DVT, deep vein thrombosis; HM, hand motions; HTN, hypertension; IHD, ischemic heart disease; LI, laser iridotomy; LL, lymphoplasmacytic lymphoma; LP, light perception; MH, medical history; MVR, mitral valve replacement; NLP, no light perception; OH, ocular history; PE, pulmonary embolus; PEA+IOL, phacoemulsification and intraocular lens; PI, peripheral iridectomy; SO, silicone oil; VHC, viral hepatitis C.

**Table 2:** Summary of the 30 cases from Table 1, divided into the nAMD and non-nAMD groups.

	**N**	**Age**	**Female**	**IOP**	**Initial ** **V** **A**≦**HM**	**Final ** **V** **A**≦**HM**
Total cases	30	72.3 ± 14.0	18 (60%)	58	80%	86%
nAMD	17	76.7 ± 10.4	9 (53%)	58.2	100%	100%
Non-nAMD	13	67.4 ± 17.2	9 (69%)	57.6	58%	67%

## Data Availability

Data sharing not applicable to this article as no datasets were generated or analyzed during the current study.
